# Exercise reduces the anxiogenic effects of meta-chlorophenylpiperazine: The role of 5-HT2C receptors in the bed nucleus of the stria terminalis

**DOI:** 10.3389/fnsyn.2022.1067420

**Published:** 2023-01-13

**Authors:** James H. Fox, Melissa N. Boucher, Khalil S. Abedrabbo, Brendan D. Hare, Bethany A. Grimmig, William A. Falls, Sayamwong E. Hammack

**Affiliations:** Department of Psychological Science, University of Vermont, Burlington, VT, United States

**Keywords:** anxiety, serotonin, extended amygdala, stress, wheel running

## Abstract

**Introduction:**

Two weeks of voluntary exercise in group-housed mice produces a reduction in anxiety-like behaviors across a number of different measures, including a reduction in the anxiety levels typically produced by the anxiogenic serotonergic drug m-chlorophenylpiperazine (mCPP), an agonist at 5-HT2C/2b receptors. We have previously demonstrated that 2-weeks of voluntary exercise blunted the anxiogenic effects of systemic mCPP, and we have also shown that mCPP infused into the bed nucleus of the stria terminalis (BNST) is anxiogenic. Here we follow up on these reports.

**Methods:**

In Experiment 1 we infused several doses of mCPP into the BNST with or without the 5-HT2C antagonist SB242084. In Experiment 2, we administered mCPP into amygdala subregions and the dorsal hippocampus to investigate site specificity. In Experiment 4 we lesioned the BNST and subsequently infused mCPP systemically, and in Experiment 4 we used RNAscope^®^ to assess BNST 5-HT2C transcripts following wheel running.

**Results:**

BNST mCPP infusion increased acoustic startle responding, which was by 5-HT2C antagonism, while neither mCPP infused into the amygdala nor hippocampus was anxiogenic. Lesions of the BNST prevented the anxiogenic effect of systemically administered mCPP. Lastly, exercise reduced 5-HT2C transcripts in the BNST.

**Discussion:**

These results suggest that the BNST is a critical site of action for the effects of exercise on mCPP. Together these data suggest that exercise may reduce 5-HT2C receptor function in the BNST, which may, in part, explain some of the anxiolytic effects associated with wheel running.

## Introduction

Substantial evidence has suggested that voluntary exercise can markedly reduce anxiety and improve both pharmacological and behavioral treatment outcomes. For example, exercise has been associated with improvement in treatment outcomes for both depression and anxiety (Byrne and Byrne, 1993; Salmon, 2001; Brosse et al., 2002) and may be particularly effective in managing post-traumatic stress disorder (PTSD; Broocks et al., 1999). Exercise is extremely anxiolytic in rodents across a variety of behavioral tests (Dishman et al., 1996; Binder et al., 2004; Salam et al., 2009); we have shown that 2 weeks of voluntary exercise produces a robust anxiolytic effect in C57BL/6J mice, as evidenced by a reduction in startle amplitude, increased time spent in the center of an open field, decreased stress-induced hyperthermia, and increased social interaction (Salam et al., 2009).

In addition to reducing behavioral indices of anxiety, voluntary exercise has been shown to confer resistance to subsequent stressor exposure. Hence, several weeks of voluntary exercise can block the behavioral changes (including increased anxiety-like behavior) associated with inescapable shock in rats (Greenwood et al., 2003) and attenuates stress-induced hyperthermia in mice (Salam et al., 2009). Moreover, we have previously shown that exercise blunts the anxiogenic effect of systemic injections with the serotonin (5-HT) agonist, meta-chlorophenylpiperazine (mCPP; Fox et al., 2008). Together these data suggest that voluntary exercise can both reduce basal levels of anxiety, and the negative impact of anxiogenic challenges.

The anxiolytic effects of exercise are likely mediated by central systems associated with coordinating stress responses and anxiety-like behavior. In particular, the bed nucleus of the stria terminalis (BNST) has recently been shown to represent the primary relay by which limbic activation modulates peripheral stressor responding at the level of the hypothalamic paraventricular nucleus (PVN, Herman et al., 1994; Makinson et al., 2015). Hence, BNST activity can both excite and inhibit PVN responding (Choi et al., 2007), and is required for PVN modulation by the hippocampus and medial prefrontal cortex (Radley and Sawchenko, 2011). In addition to its crucial role in mediating stress responses, the BNST has also been argued to mediate anxiety-like behavior in a number of paradigms in rodents (Waddell et al., 2006; Walker et al., 2009; Goode et al., 2019, 2020), non-human primates (Kalin et al., 2005) and humans (Somerville et al., 2010). Interestingly, voluntary exercise has been shown to reduce BNST c-fos expression after uncontrollable shock (Greenwood et al., 2005), as well as increase the expression of glutamic acid decarboxylase-67 (GAD67), which is consistent with a reduction in BNST activity and reduced anxiety-like behavior.

BNST activity is tightly modulated by 5-HT input originating from the stress-responsive caudal dorsal raphe nucleus (DRN; Levita et al., 2004; Guo et al., 2009, see Hammack et al., 2009 for review), where different responses of BNST neurons to 5-HT are mediated by multiple 5-HT receptor subtypes, so that the hyperpolarization response appears to be mediated by G_i_-coupled 5-HT1A receptors, whereas the depolarization response can be mediated by the G_q_-coupled 5-HT2A and 5-HT2C receptors. In support, systemic injection of the 5-HT agonist, mCPP, which has a high affinity for 5-HT2B and 5-HT2C receptors, increases BNST c-Fos expression and anxiety-like behavior, and the BNST may be the critical site of action for these effects (Singewald et al., 2003). Based on these studies, increasing 5-HT2C activation in the BNST should be anxiogenic.

Consistent with a critical role for BNST 5-HT2C activation in emotion-related responding, Marcinkiewcz et al. (2016) demonstrated that serotonin release from dorsal raphe afferents in the BNST acts on 5-HT2C receptors to excite a subpopulation of corticotropin-releasing factor (CRF) neurons to promote anxiety-like behavior, and also showed that 5-HT2C receptors in the ventral BNST may mediate anxiety-related behaviors during alcohol withdrawal (Marcinkiewcz et al., 2016). Moreover, Pelrine et al. (2016) also demonstrated that BNST 5-HT2C receptors mediate the effects of the selective serotonin reuptake inhibitor citalopram when delivered immediately before fear conditioning (Pelrine et al., 2016). Together these data suggest that BNST 5-HT2C receptors play a critical role in mediating behaviors related to emotional processing.

Exercise has been shown to modulate the activity of several neurotransmitter systems, including norepinephrine, GABA, and serotonin (Chaouloff, 1989; Dishman, 1997; Greenwood et al., 2003, 2005; Hill et al., 2010). Marathon runners or humans placed on a 10-week exercise regimen and subsequently challenged with the 5-HT agonist mCPP exhibited a decreased cortisol response as compared to healthy, non-exercising controls (Broocks et al., 1999, 2001). These results suggested that exercise decreased the function of 5-HT2B/C receptors, since the cortisol response to the 5-HT1A agonist ipsaprone was not different between exercising and non-exercising groups (Broocks et al., 1999, 2001). We have found that changes in baseline acoustic startle responding represent a reliable behavioral response that is sensitive to BNST serotonin manipulations (Levita et al., 2004; Guo et al., 2009), and this response is enhanced by intra BNST mCPP administration (Marcinkiewcz et al., 2016). Consistent with these data, we have shown that 2 weeks of voluntary exercise blocks the enhanced startle responding observed after systemic mCPP injection suggesting that central 5-HT2B and/or 5-HT2C receptors are desensitized/downregulated (Fox et al., 2008). The brain region/s mediating the anxiogenic effects of mCPP and their modulation by exercise is/are unknown. Hence, the following series of experiments were designed to determine whether reduced BNST 5-HT2C receptor function mediates the effects of voluntary exercise on the anxiogenic effects of mCPP. Our results suggest that altered responding of the BNST to 5-HT may underlie the anxiolytic and stress-resistance effects of voluntary exercise.

## Methods

### Animals

Eight week old, male C57BL/6J mice were obtained from Jackson Laboratories in Bar Harbor, Maine (For Experiment 5, both male and female 12 week old C57BL/6J mice were used). Mice were housed in groups of four in standard acrylic cages [24 cm (W) × 45 cm (D) × 20 cm (H)] located in an Association for Assessment and Accreditation of Laboratory Animal Care (AAALAC) approved conventional animal facility. Mice were maintained on a 12 h light/dark cycle (lights on at 07:00 h) with food and water available at all times. A 7-day acclimation period was given to mice after their arrival before introduction of the running wheels. All procedures were approved by the University of Vermont Animal Care and Use Committee.

### Exercise

Mice were given ad lib access to a running wheel (Superpet mini run-a-round, measuring 11.4 cm in diameter) for 2 weeks prior to the start of behavioral testing. For half of the cages, the wheels were locked preventing running (non-exercising control) and for the remaining cages the wheels were functional. Salam et al. (2009) found that a cage of four mice shared the running wheel (Salam et al., 2009). In that study, each animal contributed to an average of 25% of the total distance of ~18 kilometers per cage per 24 h recorded (with a range of 16–34%), and there was no relationship between distance-run and startle responding (Salam et al., 2009). We did not record individual running in the current study, but no differences in weight between the non-exercising and exercising mice were found.

### Surgical procedures

Cannulae (22 gauge inner diameter) were obtained from Plastics One (Roanoke, VA). Mice were anesthetized using 2% Isoflurane and oxygen and then placed into a stereotaxic instrument (Steolting, Wood Dale, Illinois). The scalp of the mice was shaved and then scrubbed in alternate with 9% betadine and 95% ethyl alcohol. The scalp was opened using a cut along the midline and then the skull lightly scraped with the edge of a scalpel blade to remove any membrane material. A small burr hole was drilled in the skull where the cannulae were lowered. BNST coordinates were 0.3 mm anterior to Bregma, 2.6 mm lateral, and 4.2 mm ventral (coordinates for control sites are specified below). The cannulae were lowered at a 20 degree angle in order to avoid hitting the ventricles which lie dorsal and medial to the BNST (Levita et al., 2004). The same procedure was done for both the left and right BNST. After lowering both cannulae, they were affixed to the skull using glue (Loctite 454, Locktite, Westlake, OH) and a glue hardening accelerator (Loctite 7542). Mice were given 0.05 mg/kg of buprenorphine prior to being removed from the sterotaxic apparatus. The mice were allowed to recover under a heat lamp prior to being returned to their home cage and the colony room. Mice were monitored daily and received 3 more doses of buprenorphine to help alleviate pain associated with the surgical procedure.

For BNST lesions, surgical procedures were similar to those described for implanting cannulas. The lesion sites were targeted at 0.3 mm anterior to Bregma, 2.6 mm lateral, and 4.2 mm ventral. After being placed in the ear bars, the head was shaved and cleaned with an alcohol-betadine wash, the scalp was incised and retracted. Small burr holes were drilled through the skull above the lesion site. A 0.5 μl airtight glass syringe (Hamilton, Reno, Nevada) mounted on a motorized stereotaxic injector (Nano Injector, Steolting, Wood Dale, Illinois) was lowered into the injection site. NMDA (0.2 μl of 20 μg/ul NMDA) was then infused at a rate of 0.05 μl/min. The injector was left in for an additional 2 min to allow diffusion of the drug. Metal suture clips were used to close the scalp. Postoperative procedures were as described for cannulations. Amygdala lesions implemented the same methods, except coordinates were −1.7 mm posterior, 3.0 mm lateral, and −4.3 mm ventral to bregma, and for the hippocampus, two bilateral cannula were implanted with the following coordinates, −1.9 mm posterior, 1.5 mm lateral, and −2 mm ventral to bregma, and −2.4 mm posterior, 1.5 mm lateral, and −2 mm ventral to bregma.

### Drug infusions and acoustic startle measurement

For Experiment 1 and 2, mice were infused with mCPP HCl (Tocris, Ellisville, MO) into the BNST, amygdala or hippocampus, which was mixed fresh the morning that the behavioral testing took place. For mCPP infusion, mice were gently restrained and the stylets removed from the guide cannulae prior to insertion of the internal cannulae. Internal cannulae were connected using polyethelene tubing to a 10 μl micro syringe (Hamilton, Reno, NV). Bilateral infusions of 0.1, 1.0, or 10 μg mCPP or equivolume (0.5 μl) artificial cerebrospinal fluid vehicle, were made using a mechanical infusion pump (KD Scientific, Holliston, MA) a rate of 0.25 μl/min for 2 min. Internal cannulae were left in place for an additional 2 min to aid in diffusion of the drug into the target area. Also in Experiment 1, 0.1 μg of the 5-HT2C antagonist SB242084 (Tocris, Ellisville, MO) was mixed with 1 μg or 10 μg mCPP in dH20 vehicle, and infused into the BNST as described above. We have previously shown that infusion of dH2O vehicle does not alter behavior (Hammack et al., 2003).

### Acoustic startle measurement

As noted above, while we have demonstrated that exercise is anxiolytic across multiple behavioral tests in mice, changes in baseline acoustic startle responding represent a robust and reliable behavioral measure that can be observed across species that can also be used to assess both anxiolytic and anxiogenic effects of BNST serotonin manipulation (Guo et al., 2009; Marcinkiewcz et al., 2016). Moreover, we have observed significant enhancement in baseline startle responding following both systemic and intra-BNST mCPP administration (Fox et al., 2008; Marcinkiewcz et al., 2016); hence, acoustic startle responding was implemented as the primary behavioral measure in the present report. The startle tests were conducted in eight sound attenuating cubicles measuring 58 cm (W) × 32 cm (D) × 55 cm (H). Each cubicle was lined with black, sound absorbing foam with no internal source of light. Each cubicle contained a stabilimeter device consisting of a load cell platform onto which the behavioral chamber was mounted (MED-ASR-PRO1, Med-Associates, Georgia, VT). The chamber was constructed of clear acrylic, cylindrical in shape, 12.5 cm in length, with an inner diameter of 5 cm. The floor of the chamber consisted of a removable grid composed of six steel rods 3.2 mm in diameter and spaced 6.4 mm apart. Startle responses were detected by the load cell, amplified, and digitized over a range of 0–4,096 units. Startle amplitude was defined as the largest peak to trough value within 100 ms after the onset of the startle stimulus. After a 5-min acclimation period, mice were presented with the first of 30 startle stimulus alone trials. The startle stimulus was comprised of white noise bursts lasting for 20 milliseconds. Ten stimuli of each intensity level (95, 100, and 105 dB) were presented in a pseudo-random order (the constraint being that each intensity occur within each block of three trials) with an inter-trial interval (ITI) of 60 s. The ITI ranged from 30 to 90 s. Data collection and the control and sequencing of all stimuli were controlled by Med-Associates startle reflex hardware and software.

### Histology

Following behavioral testing, mice were euthanized using pentobarbital (SleepAway, Fort Dodge Drug Company, Fort Dodge, IA), and perfused transcardially using 0.9% saline followed by 10% neutral buffered formalin. Brains were saved in 10% formalin and sectioned on a cryostat at 50–60 μm. Slices were stained with cresyl violet for cannula placement verification.

### Analysis and statistics

Mean startle amplitude will be computed for each startle stimulus intensity for each mouse and subject to ANOVA with startle intensity as a within subject variable and drug, dose, and/or exercise group as between subject variables. The criterion for significance will be set at *p* < 0.05. Significant interactions or main effects were followed up by using Tukey's HSD.

#### Experiment 1: BNST mCPP dose response and 5-HT2C antagonism

As noted above, systemic injection of mCPP has been shown to be anxiogenic in humans (Broocks et al., 1999, 2001) and rodents (Fox et al., 2008), and increases c-Fos expression within stress- and anxiety-related brain regions such as the BNST (Singewald et al., 2003); hence, mCPP may directly activate BNST neurons to increase anxiety-like behavior and/or mCPP may activate the BNST indirectly, through actions in another anxiety-related brain region. Consistent with this, we have previously reported that intra-BNST mCPP administration is anxiogenic (Mazzone et al., 2018). Moreover, mCPP is a 5-HT 2B/C agonist with lower affinity for 5-HT2A, 5-HT1B, and 5-HT1A receptors (Hoyer et al., 1994), and with the highest affinity for 5-HT2C receptors. The anxiogenic effects of mCPP have been blocked using selective 5-HT2C antagonists (Campbell and Merchant, 2003; Wood, 2003), and 5-HT2C antagonists have been used effectively in rodent models of anxiety to decrease anxiety-like behaviors (Griebel et al., 1997). Further, data suggest there may be few if any 5-HT2B receptors are centrally located (Pompeiano et al., 1994; Guo et al., 2009). In addition, activation of the 5-HT2C receptors by 5-HT results in depolarization of BNST neurons suggesting that these receptors are involved in BNST activation (Guo et al., 2009). Experiment 1 was designed to determine whether 2 weeks of prior access to an unlocked running wheel would block the anxiogenic effects of BNST mCPP at multiple doses, and also whether the effects of intra BNST mCPP could be blocked with BNST 5-HT2C antagonism. For Experiment 1, we assessed the behavioral effects of BNST-infused mCPP (0, 0.1, 1, or 10 μg per side) in mice that had access to a locked or unlocked running wheel for 2 weeks prior to surgery. One week after delivery, a locked or unlocked running wheel was introduced into the home cage and remained there for the duration of the experiment. After 2 weeks, mice were cannulated into the BNST as described above. One week later, following a baseline startle test, mice were matched carefully as to not confound cage by drug (so at least one of four mice in a cage had vehicle). Mice were infused into the BNST with one of the doses of mCPP or equivolume (0.5 μl per side) vehicle co-administered with either the 5-HT2C antagonist SB242084 (0.1 μg/0.5 μl) or vehicle and the acoustic startle response was assessed as described above. After behavioral testing, mice were euthanized and their brains processed for verification of cannula placement.

#### Experiment 2: Amygdala and hippocampus control sites

Experiment 2 was designed to determine if mCPP infusion into other fear- and anxiety-associated brain regions would also increase acoustic startle responding as observed in the BNST. For example, 5-HT2C receptors are expressed in the basolateral amygdala (BLA), and their activation is necessary for the anxiogenic consequences of inescapable shock (Christianson et al., 2010), while 5-HT modulation of the adjacent central amygdala (CeA) has also been associated with anxiety-like behavior. Similarly, several studies have shown that the modulation of 5-HT activity within hippocampal subregions can modulate anxiety-like behavior (see Bombardi et al., 2021 for review).

For Experiment 2, we infused mCPP (10 μg per side) or equivolume (0.5 μl per side) vehicle into the amygdala (while we targeted the central amygdala, but it is likely that the infusion spread throughout the entire extent of this structure), the hippocampus (similarly, we targeted the dorsal aspect of the hippocampus with two separate cannula for these studies). For amygdala infusions of mCPP, all procedures were identical to Experiment 1, except that for these control site studies, mice were not given access to an unlocked running wheel (we did not assess the effects of exercise in these studies).

Similarly, for hippocampal infusions of mCPP, all procedures were identical to Experiment 1, except that mice were not given access to an unlocked running wheel.

#### Experiment 3: BNST lesions and mCPP

In Experiment 3, we investigated the anxiogenic effects of systemically administered mCPP following an excitotoxic lesion of the BNST using *N*-Methyl-D-aspartic acid (NMDA). Using an excitotoxic lesion would allow for direct inactivation of the BNST while sparing fibers (Brace et al., 1997; Hammack et al., 2015). If the BNST is important for the anxiogenic properties of mCPP and not another area, then the BNST lesion should block the anxiogenic effects of systemic mCPP.

One week following BNST lesion or sham surgeries (described above), A baseline startle response was collected after surgery and then all mice were matched for drug group (1 mg/kg or vehicle) for the first day of testing. Matching consisted of making the overall mean acoustic startle responses for each drug group as close to equal as possible by sorting the means for each animal into each drug group. Thus, the overall mean acoustic startle response means were not different for either the vehicle or mCPP groups prior to being tested. The mice were then retested 48 h after being tested with vehicle or mCPP with their drug group crossed over so that all animals received both mCPP and vehicle. Mice were given 15 min after i.p. injection prior to being tested for startle.

#### Experiment 4: Exercise and 5-HT2C transcripts

In Experiment 4, we implemented RNAscope^®^ strategies to investigate 5-HT2C transcripts in the BNST following wheel running. Male and female mice were given access to a locked or unlocked running wheel in their home cage. Following 2 weeks of running or control treatment, mice were deeply anesthetized and rapidly decapitated. Brains were flash frozen, and sectioned on a cryostat at 10 μm, and thaw mounted on SuperFrostPlus Slides. Sections were then fixed in 4% paraformaldehyde prior to treatment with increasing concentrations of ethanol per the Advanced Cell Diagnostics RNAscope^®^ Fluorescent Multiplex Reagent Kit (Cat 20850) protocol. Sections were hybridized to 5-HT2C mRNA probe (Cat 401001) and then amplified using the RNAscope^®^ Fluorescent Multiplex Reagent Kit. Slides were stained with DAPI and cover-slipped with Citifluor Antifadent mounting media.

Slices were imaged at 40X on an Olympus fluorescent microscope (Olympus Corporation of the Americas, Center Valley, PA, USA). Images were preprocessed using Fiji, merging the DAPI (blue) and 5-HT2C mRNA puncta (green) channels. Images were then analyzed using CellProfiler (Broad Institute). Analysis of mRNA puncta per cell was carried out as described by Erben and Buonanno (2019). Images were converted to greyscale and nuclei and puncta were identified by their size. Prior to analysis of all images, a scorer blind to condition manually counted cells and confirmed accuracy of the CellProfiler pipeline used.

## Results

### Experiment 1: BNST mCPP dose response and 5-HT2C antagonism

**Figure 1:**
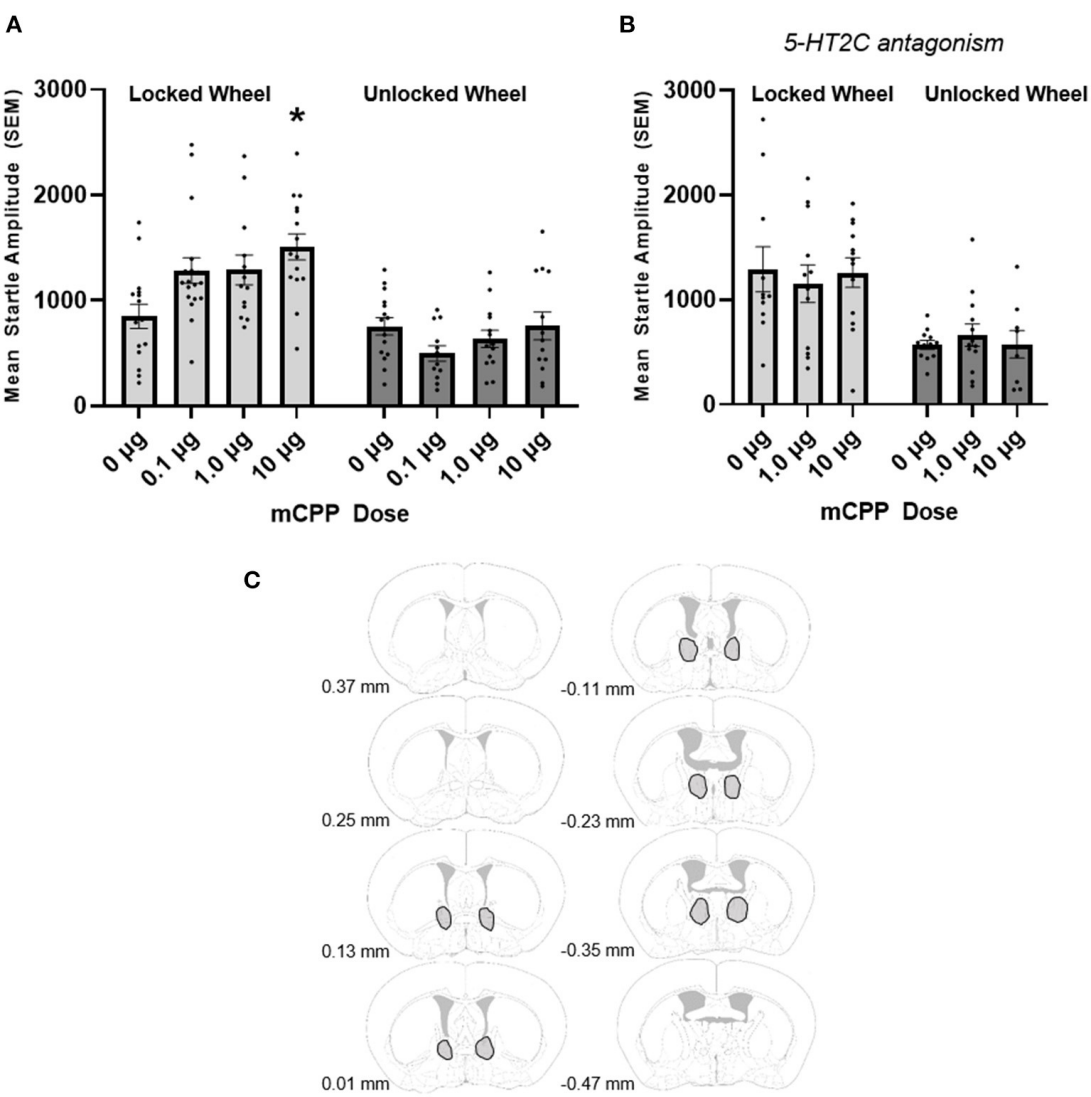
**(A)** mCPP increases the acoustic startle response in non-exercising (locked wheel) mice and exercise (unlocked wheel) blocks this effect. ANOVA revealed a significant interaction between running wheel condition and dose of mCPP as well as significant main effects for running wheel group and dose. Non-exercising mice showed significant increases in the acoustic startle response with the 10 μg dose of mCPP infused into the BNST (denoted by an asterisk). In contrast, the exercising mice did not show any significant effect on the acoustic startle response with any dose. **(B)** The 5-HT2C antagonist SB242084 blocks the potentiation of the startle response in non-exercising mice. ANOVA was not significant and there was no effect of mCPP when co-infused with SB242084 into the BNST. **(C)** Outlined in gray are BNST regions in which cannula tips deemed acceptable for analysis were located. Images were adapted from Süß et al. (2022).

As noted above, Experiment 1 was designed to determine whether 2 weeks of prior access to an unlocked running wheel would block the enhancement of baseline acoustic startle produced by BNST mCPP at multiple doses. Only animals with cannula placements into the BNST were included in our analyses, and there were 185 mice used in Experiment 1. Figure 1C depicts the BNST regions containing injection sites included for analyses using figures modified from Paxinos and Franklin (2019).

For data in which mCPP was successfully infused into the BNST, using a between subjects 2 × 4 ANOVA (exercise group × 4 doses of mCPP) we found a significant interaction between exercise group (non-exercising or exercising) and dose of mCPP [*F*_(3, 107)_ = 4.156, *p* = 0.008]. There were also main effects for both the exercise group [*F*_(1, 107)_ = 50.49, *p* < 0.0001] and dose of mCPP [*F*_(3, 124)_ = 3.076, *p* = 0.031; see Figure 1A].

Data were further analyzed using one-way ANOVAs for the exercising and non-exercising groups separately in order to further investigate how the doses of mCPP were affecting the acoustic startle response. As can be seen in Figure 1A, a significant effect of dose was found [*F*_(3, 537)_ = 4.788, *p* = 0.0015] in the non-exercising mice. For the locked wheel groups, Tukey's *post-hoc* analyses revealed an increase in the acoustic startle response for the 10 μg mCPP (but not other doses; nor did other doses differ from the others in this *post-hoc* test) when compared to mice who received vehicle. In contrast, the mice who had an opportunity to run for at least 2 weeks on a functional running wheel did not show a significant effect of dose on the acoustic startle response [*F*_(3, 50)_ = 1.583, *p* = 0.2051]. The mean acoustic startle response in exercising mice remains relatively unchanged with all three doses of mCPP compared to the mice who received vehicle.

Notably, in this study we had a number of missed cannula over the course of the very large experiment; hence, a total of 50 mice were not included for analyses because one or both of the injections sites landed outside of the region of the BNST (these misses were variable in their location, and included ventricular injections). Because these misses were reasonably distributed across eight experimental groups, we conducted an analysis to determine whether mCPP infusion outside of the BNST elevated startle. We found that there was no effect of mCPP on acoustic startle responding when mCPP was infused in regions outside of the BNST [*F*_(3, 40)_ = 0.3046, *p* = 0.8219; data not shown] nor was there an interaction between missed mCPP infusions and exercise [*F*_(3, 40)_ = 0.4806, *p* = 0.6976; data not shown]. Exercise was generally anxiolytic, reducing acoustic startle across missed mCPP doses [*F*_(1, 40)_ = 4.172, *p* = 0.0477; data not shown].

We also asked whether the effects of intra-BNST mCPP could be abrogated by the 5-HT2C antagonist SB242084. Only mice with cannula placements in the BNST were used in the analysis. Eighteen mice were removed due to misses. We did not analyze data from the missed BNST animals separately because both mCPP and SB242084 would have been infused outside of the BNST. SB242084 attenuated the anxiogenic effect of intra BNST mCPP. From a between subjects 2 (exercise group) × 3 (drug) ANOVA, no interaction between exercise and drug was found [*F*_(2, 64)_ = 0.3669, *p* = 0.6943; Figure 1B]. There was a main effect of exercise [*F*_(1, 64)_ = 28.16, *p* < 0.0001] but no effect of drug [*F*_(2, 64)_ = 0.01293, *p* = 0.9872]. This is consistent with the previous experiments showing higher startle amplitudes in non-exercising mice.

Interestingly, 5-HT2C antagonism appeared to enhance acoustic startle responding in non-exercising mice. A two-way ANOVA examining the effects of SB242084 treatment with exercise (at the zero mCPP dose) revealed a significant effect of exercise [*F*_(1, 49)_ = 11.10, *p* = 0.0016] and a significant interaction between SB242084 treatment and exercise [*F*_(1, 49)_ = 6.469, *p* = 0.0142]. The reason for this anxiogenic effect of SB242084 in sedentary mice is unclear, and may result from complexities in the serotonin system and/or complexities in the pharmacological actions of SB242084. We discuss these possibilities below, and acknowledge that these observations suggest caution when interpreting these pharmacological studies.

### Experiment 2: Amygdala and hippocampus control sites

**Figure 2:**
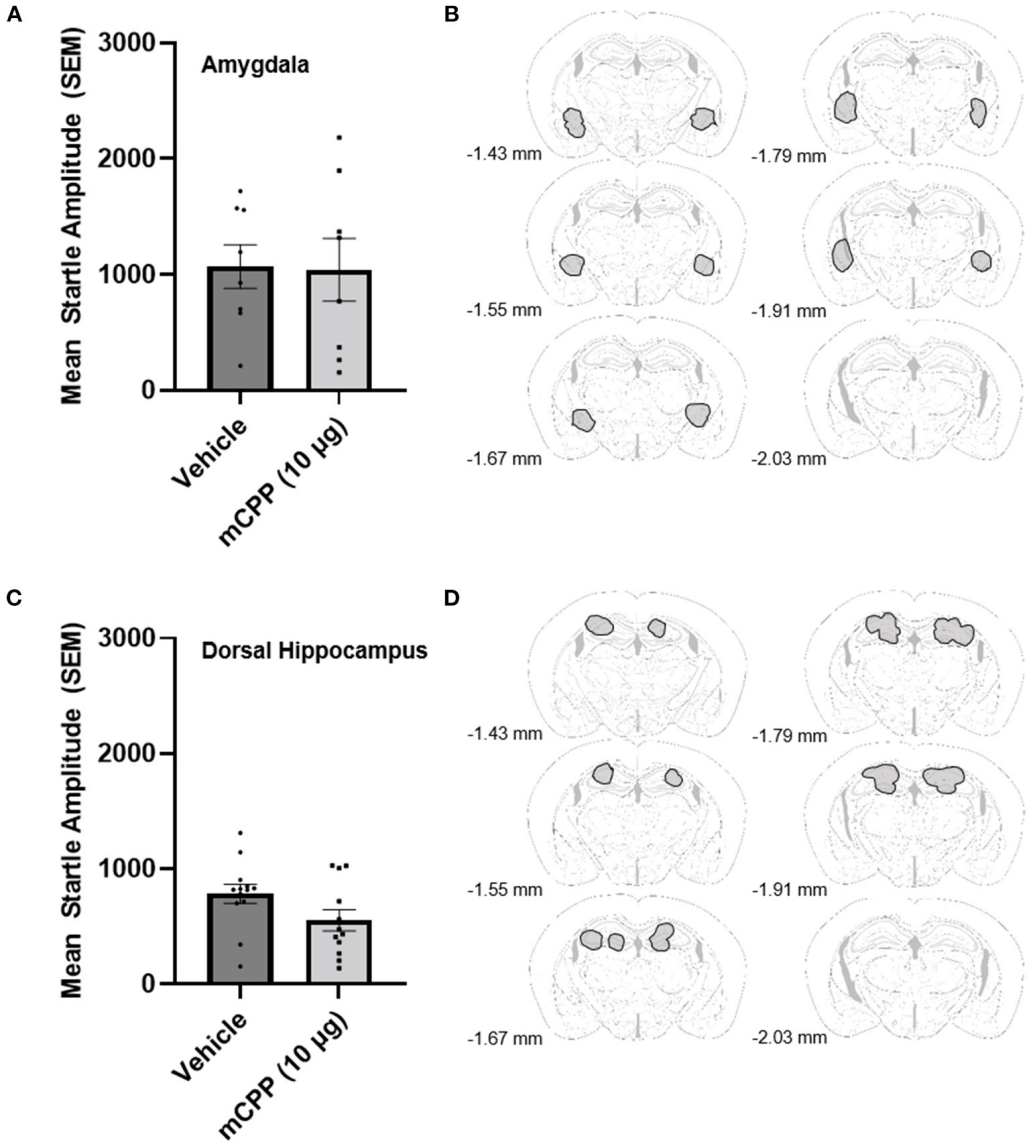
**(A)** mCPP does not potentiate the acoustic startle response when directly infused into the amygdala. Unpaired *t*-test did not show a significant effect of 10 μg of mCPP on the acoustic startle response in non-exercising mice animals. **(B)** Outlined in gray are amygdala regions in which cannula tips deemed acceptable for analysis were located. **(C)** mCPP does not potentiate the acoustic startle response when infused into the dorsal hippocampus. Unpaired *t*-test did not show a significant effect of 10 μg of mCPP on the acoustic startle response in non-exercising mice. **(D)** Outlined in gray are hippocampal regions in which cannula tips deemed acceptable for analysis were located. Images were adapted from Süß et al. (2022).

Experiment 2 was designed to determine whether mCPP infusion into the region of the amygdala or hippocampus was anxiogenic. For amygdala infusions, data from 16 mice were analyzed and placements in the amygdala were closer to the CeA rather than the BLA but could have included both areas as it appears that placements were slightly medial and dorsal to the BLA. One animal was removed because placements were too dorsal and outside the area of the amygdala, and there were no missed cannula for the hippocampal cannulations, where data from 25 mice were analyzed. An unpaired *t*-test revealed that 10 μg of mCPP into the amygdala did not significantly potentiate the acoustic startle response over vehicle infusion [*t*_(14)_ =0.08641, *p* > 0.9324]. Figures 2A, B shows the data for the intra amygdala infusion of vehicle or 10 μg of mCPP. An unpaired *t*-test also revealed that 10 μg of mCPP infused into the hippocampus similarly did not increase acoustic startle [*t*_(23)_ = 1.877, *p* = 0.0733; Figures 2C, D], although it is notable that there was a trend toward an anxiolytic effect (reduced startle responding). These data suggest that neither amygdala nor hippocampal subregions likely mediated the anxiogenic effects (enhanced acoustic startle responding) of mCPP.

### Experiment 3: BNST lesions and mCPP

In Experiment 3 we investigated whether BNST excitotoxic lesions could attenuate the anxiogenic response of systemic mCPP administration. Histological analyses of the lesion sites demonstrated lesions of the BNST in all eight mice receiving BNST NMDA, and data from all mice were included in our analyses. Data from 21 total mice were analyzed.

**Figure 3:**
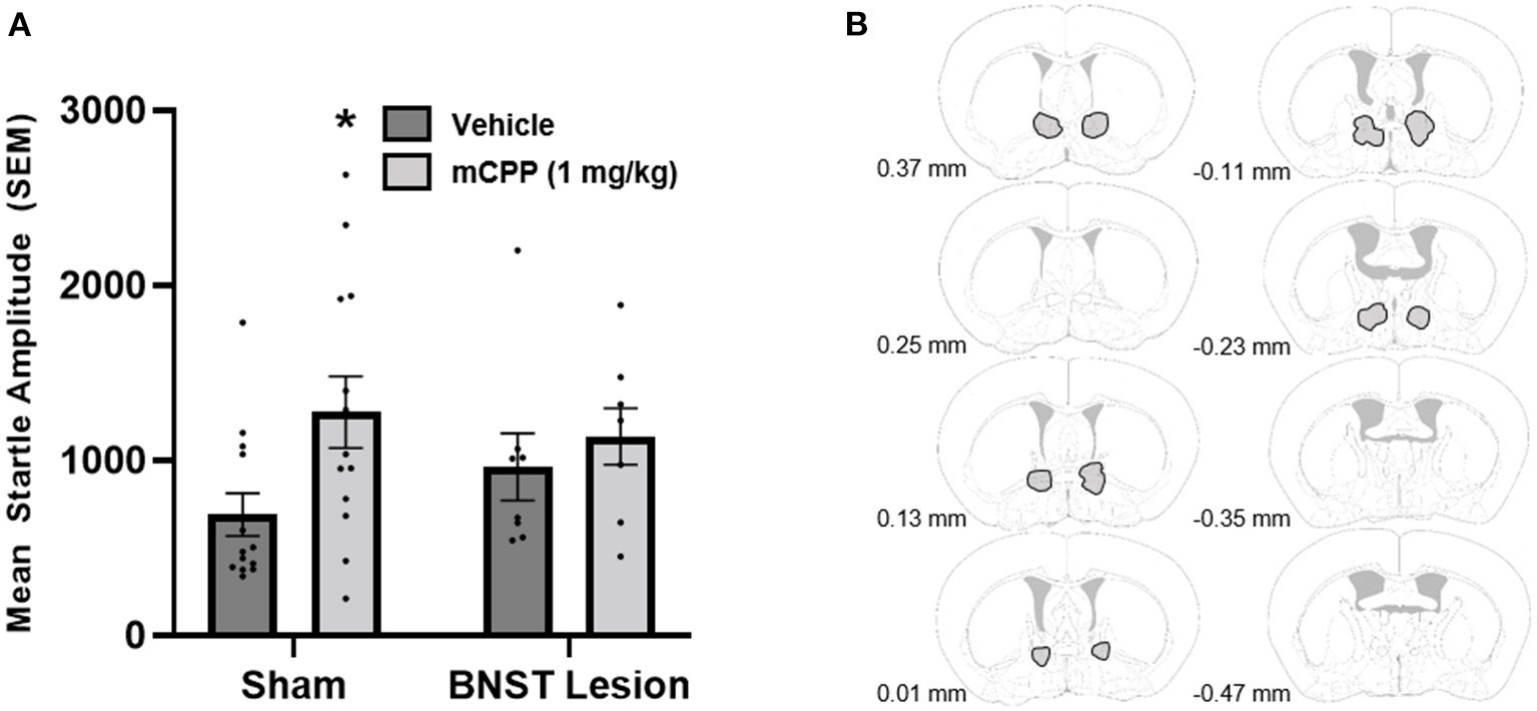
**(A)** BNST lesions made with NMDA blocked mCPP induced potentiation of the acoustic startle response. Sham animals showed a significant increase in their startle response when administered 1 mg/kg of mCPP i.p. (denoted by an asterisk). **(B)** Outlined in gray include all of the regions in which NMDA lesions extended in the analysis. Images were adapted from Süß et al. (2022).

Data showed that mCPP did not increase startle in mice that had lesions of the BNST, and mice that had sham lesions showed an increase in their anxiety-like behavior following injection with mCPP (Figure 3). A mixed ANOVA (lesion status was between subject and mCPP injection was within subject) showed a significant effect of drug [*F*_(1, 19)_ = 11.56, *p* < 0.0030] and a trend for a drug by lesion group interaction [*F*_(1, 19)_ = 3.42, *p* = 0.0799]. No between-group effect of lesion or sham was observed. While it looks like the BNST lesioned vehicle group was elevated as compared to vehicle shams *post-hoc* unpaired *t*-tests revealed no difference [*t*_(19)_ = 1.2262, *p* = 0.2223], nor was there a difference in mCPP treatment groups [*t*_(19)_ = 0.4738, *p* = 0.6410]. Nevertheless, from a visual inspection of the data, it could be argued that the lack of effect of mCPP in the BNST lesion could result from higher baseline startle values in the vehicle group. While we acknowledge this possibility, it is notable that these values are not near ceiling startle values for mice in our apparatus. Moreover, these data in combination with Experiments 1 and 2 provide a more compelling argument that the BNST represents an important site of action for these effects, than either experiment provides on its own. Because the interaction value suggested a strong trend, and we made an a priori prediction that BNST lesions would prevent an anxiogenic effect of mCPP, we conducted *post-hoc* paired *t*-tests to investigate whether an anxiogenic effect of mCPP could be observed within sham and BNST lesions groups. These *post-hoc* paired *t*-tests revealed that mice that received sham lesions showed a significant increase in their startle response when injected with 1 mg/kg of mCPP compared to vehicle [*t*_(24)_ = 2.443, *p* = 0.0223]. In contrast, the mice with lesions of the BNST did not show a significant increase in their startle response when they were injected with mCPP compared to when they were injected with vehicle [*t*_(14)_ = 0.6868, *p* = 0.14].

### Experiment 4: Exercise and 5-HT2C transcripts

**Figure 4:**
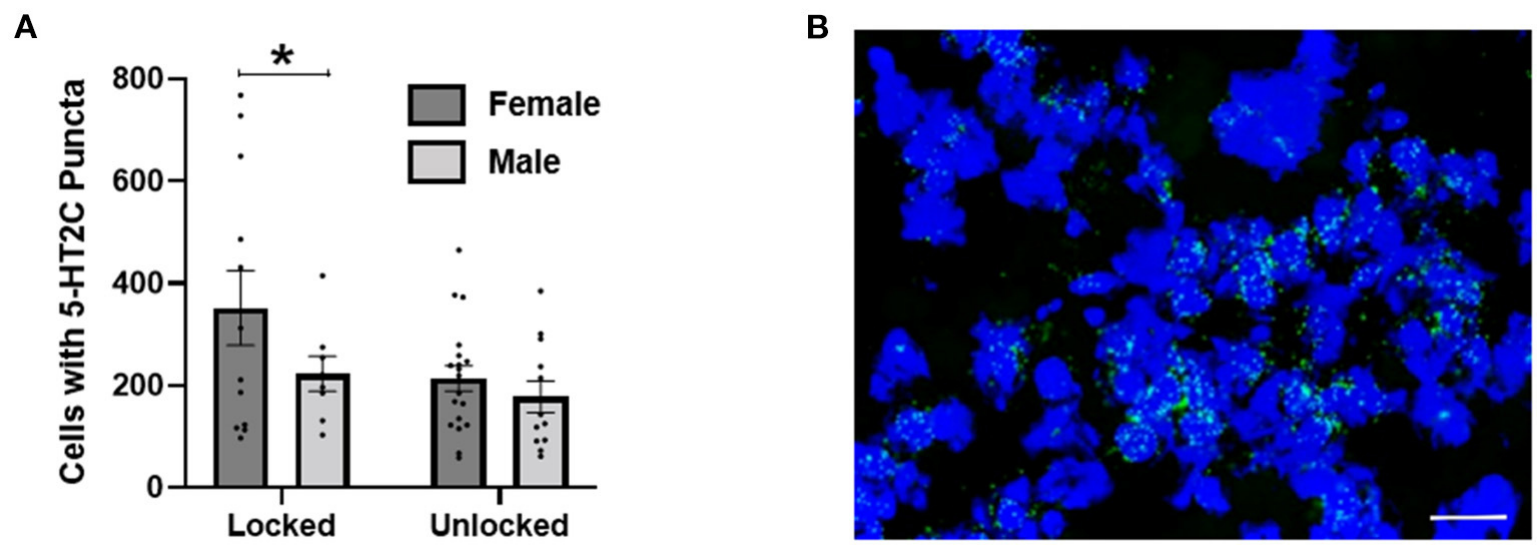
**(A)** Using RNAscope^®^, 2 weeks of exercise (unlocked wheel) reduced 5-HT2C transcripts in the BNST. Two-way ANOVA revealed a main effect of exercise (denoted by an asterisk), but no effect of sex or interaction. **(B)** An example of 5-HT2C puncta observed using RNAscope^®^. Scale bar = 20 μm.

In Experiment 4, we implemented RNAscope^®^ strategies to investigate 5-HT2C transcripts in the BNST following wheel running in 51 BNST tissue slices from 20 mice. Using a between subject 2 × 2 ANOVA (exercise group × sex), we found a significant main effect of exercise group [non-exercising or exercising; *F*_(1, 47)_ = 4.135, *p* = 0.048] on the total number of BNST neurons that contained at least five puncta, and a trend toward a main effect of sex [*F*_(1, 47)_ = 3.397, *p* = 0.072], but no interaction [*F*_(1, 47)_ = 1.069, *p* = 0.307]. Hence, exercise reduced 5-HT2C receptor transcripts. While we did not observe a main effect of sex nor an interaction between sex and exercise, the 5-HT2C reduction appears larger in female mice (Figure 4).

## Discussion

We have previously demonstrated that voluntary wheel running reduces the anxiogenic effects of systemic mCPP injection in mice. Here our data suggest that this effect of exercise may be mediated by a reduction in BNST 5-HT2C receptor function. In Experiment 1, we demonstrated that 2 weeks of voluntary wheel running blocks the anxiogenic effect of BNST mCPP at multiple doses, and mCPP has no anxiogenic action when administered with a 5-HT2C receptor antagonist. In Experiment 2, we demonstrated that mCPP infusion into the amygdala or hippocampus does not appear to produce an anxiogenic response using a high dose of mCPP (10 μg). We acknowledge it is possible that mCPP exhibits a different dose-response relationship in these structures as compared to the BNST; however, we observed a monotonic dose-response relationship for the anxiogenic effects of mCPP in the BNST. This monotonic relationship is consistent with the dose-response function for systemic mCPP (Fox et al., 2008); hence, these data are suggestive that the BNST is a key site of action. In Experiment 3 we demonstrated that excitotoxic BNST lesions prevent the anxiogenic effects of systemically administered mCPP. Together these studies suggest the BNST is a key site for the anxiogenic actions of mCPP and may be involved to the anxiolytic effects of exercise. In Experiment 4, we demonstrated that 5-HT2C receptor transcripts are downregulated following wheel running. Together, these data suggest that exercise may reduce BNST 5-HT2C and this may contribute to the anxiolytic effects of exercise, and the ability of prior wheel running to prevent the anxiogenic effects of mCPP. Interestingly, while we found that 5-HT2C receptor transcripts were reduced following wheel-running, it appears that this effect was primarily driven by a reduction in female mice (although there was not a significant effect across sex or interaction). A full assessment of the effects of exercise in female mice has not been conducted and is an important future study. Notably, changes in transcript levels may not reflect the number of functional receptors present on BNST neurons, and a thorough characterization of this effect in males and females is an important future goal.

Interestingly, BNST 5-HT2C antagonism did appear to have an anxiogenic effect in non-exercising mice (not treated with mCPP). The 5-HT system is complex, and BNST neurons can express multiple 5-HT receptor subtypes to regulate neuronal activity that may interact with each other in complicated ways (Hammack et al., 2009). The behavioral effects of BNST 5-HT2C antagonism could suggest 5-HT release under the testing conditions utilized, possibly associated with the stress of drug infusions, and it is possible that 5-HT2C antagonism could increase 5-HT bioavailability at other 5-HT receptor subtypes, some of which could also be anxiogenic. We acknowledge that this mechanism might also possibly explain the anxiogenic actions of BNST mCPP infusion. Moreover, these data could also be explained if SB242084 has mixed antagonist/partial agonist properties at BNST 5-HT2C receptors and/or effects at other receptor subtypes. Together these observations highlight weaknesses inherent in pharmacological strategies, and suggest further studies are needed implementing multiple strategies to explore the anxiolytic effects of exercise. Notably, other reports utilizing different approaches have demonstrated anxiogenic effects of BNST 5-HT2C receptor activation (Marcinkiewcz et al., 2016; Pelrine et al., 2016).

While these studies were focused on anterior BNST regions, the BNST subregions mediating the effects of mCPP were not delineated. There is now a substantial literature demonstrating that different BNST subregions may have opposing influences on stress- and anxiety-responding (Choi et al., 2007; Kim et al., 2013; Turesson et al., 2013). As described above, Marcinkiewcz et al. (2016) demonstrated that 5-HT2C receptors likely excite a subpopulation of CRF neurons in the region of the anterior BNST to promote anxiety-like behavior (Marcinkiewcz et al., 2016). The selective reduction of 5-HT2C expression on this subpopulation of CRF neurons would be expected to reduce anxiety-like behavior, as well as the anxiogenic effects of mCPP. However, there may also be an anxiogenic population of 5-HT2C receptors in the ventral BNST, which may mediate anxiety-related behaviors during alcohol withdrawal (Marcinkiewcz et al., 2015) that could also be reduced by prior wheel-running. That several groups have now demonstrated an anxiogenic effect of BNST 5-HT2C activation (Pelrine et al., 2016), it is not surprising that this receptor subtype might be a key target for the anxiolytic effects of wheel-running. However, the data presented here do not have the spatial resolution to determine the BNST subregion targeted.

The current studies implemented changes in acoustic startle responding as the primary measure of anxiety-like behavior because this behavior is consistent across many species while also being particularly sensitive to serotonin manipulations (Guo et al., 2009; Marcinkiewcz et al., 2016). It is unknown whether these manipulations would produce similar results with other behavioral tests of anxiety. We have previously demonstrated that 2 weeks of wheel running is anxiolytic across many different anxiety tests in mice (Salam et al., 2009), and BNST efferent projections have been argued to target many brain regions to coordinate anxiety-like behavior across several behavioral modalities (Walker et al., 2009). We acknowledge that BNST serotonin manipulations could possibly be limited to specific neuronal populations in the BNST that particularly target acoustic startle responding. Future work is needed to clarify whether these results can extend to other anxiety-like behavior modalities.

Exercise may be useful clinically. For example, exercise may shorten the amount of time necessary for the anxiolytic effects of fluoxetine, possibly by increasing the amount of extracellular 5-HT (Chaouloff, 1997). Data from our lab has shown that fluoxetine alone can blunt the anxiogenic effects of mCPP and that exercise in combination with fluoxetine may shorten the amount of time required to achieve the anxiolytic effects of fluoxetine. Bristow et al. (2000) speculate that the beneficial effects of fluoxetine given in conjunction with a 5-HT1A antagonist may be *via* a desensitization of the 5-HT2C receptors, as rats show a blunted anxiogenic response to mCPP (Bristow et al., 2000). Given that mice show a blunted response to mCPP after 2 weeks of exercise, this may also be modulated by blunting of the sensitivity of 5-HT2C receptors. Both SSRIs and exercise produce an increase in extracellular 5-HT (Chaouloff, 1997). SSRIs (Greenwood et al., 2008), mCPP (Fox et al., 2008), as well as exercise (unpublished observations) are anxiogenic acutely and all of these may be so through activation of the 5-HT2C receptors (Bagdy et al., 2001). Changes in 5-HT2C receptor activity through pre-mRNA editing because of increased 5-HT availability (Gurevich et al., 2002) may work as a feedback mechanism to control for the effects of increase 5-HT and therefore dampen the 5-HT2C receptors. This decreased activity of the 5-HT2C receptors through pre-mRNA editing may be a common mechanism through which both exercise and SSRIs are anxiolytic.

It appears that exercise may shorten the time necessary for fluoxetine to become effective at reducing anxiety possibly through a similar desensitization of 5-HT2C receptors, especially as the anxiogenic effects of antidepressant treatment have been argued to be mediated by BNST 5-HT2C activation (Pelrine et al., 2016). If this is the case, combining exercise with fluoxetine treatment may make it more likely that people will follow through with their treatment plans if it does not take as long for them to start feeling better. Treatment of anxiety disorders with CBT or a pharmacological agent is not always effective at reducing symptoms of the disorder (Katzman, 2009) and so using exercise in conjunction with these treatments or independently may help. Specifically understanding the mechanisms behind the anxiolytic effects of exercise such as reductions or decreased activity in 5-HT2C receptors may be useful in creating pharmacological agents for increasing the effectiveness of other treatment methods as well.

While exercise has been associated with both anxiolytic and antidepressant actions as noted above, the current studies implementing baseline acoustic startle responding were focused particularly on an anxiety-related behavior. While it is tempting to argue that changes in BNST 5-HT2C function may also underlie the antidepressant actions of exercise, the role of BNST activity in depression behaviors is less clear and to our knowledge BNST 5-HT2C manipulations have only been made in the context of fear- and/or anxiety-related behaviors (Levita et al., 2004; Fox et al., 2008; Guo et al., 2009; Katzman, 2009; Marcinkiewcz et al., 2015). While it may be likely that exercise effects on depression are mediated by other brain regions, further study is required to determine whether the BNST represents an important site of action.

In conclusion, 2 weeks of exercise is associated with a reduction in anxiety. The anxiogenic effects of systemic and intra BNST mCPP are blunted in mice that have exercised. The anxiogenic effect of intra BNST is mediated by 5-HT2C receptors and given that exercise blunts the effects of mCPP, and 5-HT2C receptor transcripts are reduced following 2 weeks of running, BNST 5-HT2C receptor downregulation may mediate some of the anxiolytic consequences of exercise treatment. Furthermore, the anxiogenic effects of mCPP do not appear to involve the hippocampus or the amygdala and the anxiogenic effect of systemic administration appears to be limited to the effects in the BNST. It is suggested then that exercise is anxiolytic in part through changes in the BNST.

## Data availability statement

The raw data supporting the conclusions of this article will be made available by the authors, without undue reservation.

## Ethics statement

The animal study was reviewed and approved by University of Vermont Institutional Animal Care and Use Committee.

## Author contributions

JF, WF, and SH designed the studies. JF, KA, MB, and BG ran the experiments. JF, MB, WF, and SH wrote the manuscript. All authors contributed to the article and approved the submitted version.
